# Metabolomics study of flavonoids in *Coreopsis tinctoria* of different origins by UPLC–MS/MS

**DOI:** 10.7717/peerj.14580

**Published:** 2022-12-19

**Authors:** Yi Wang, Junsen Cheng, Wei Jiang, Shu Chen

**Affiliations:** College of Horticulture and Landscape Architecture, Zhongkai University of Agriculture and Engineering, Guangzhou, China

**Keywords:** Flavonoids, *Coreopsis tinctoria*, Capitulum, Metabonomics, UPLC-QTOF-MS

## Abstract

To analyze the flavonoids in *Coreopsis tinctoria* and compare the differences in flavonoids among *C. tinctoria* of different origins, the chemical composition of *C. tinctoria* capitulum was analyzed by ultra-high-performance liquid chromatography-tandem quadrupole time-of-flight mass spectrometry (UPLC-QTOF-MS), and the flavonoid metabolites were analyzed and identified based on their retention time, mass-to-charge ratio and fragment ions in the UPLC-QTOF-MS matrix. Capitulum samples of *C. tinctoria* were collected from three locations in the Xinjiang region at different altitudes. A total of 204 flavonoid compounds were identified, and 31 different flavonoid metabolites were then identified from flowers of *C. tinctoria* of different origins. Further analysis of these 31 significantly accumulated metabolites identified seven flavonoid metabolites, namely, homoplantaginin, kaempferol, quercetin, isorhamnetin, avicularin, quercetin 3-O-(6′-galloyl)-*β*-*D*-galactopyranoside and isorhamnetin 3-O-glucoside, with high accumulation only in sample collected from Tashkurgan Tajik (TX) and low expression in sample collected from Yutian County (YT) and Shaya County (SY). Moreover, 7,4′-dihydroxyflavone and 4,4′-dimethoxychalcone showed high accumulation only in SY, and afzelin was specifically highly accumulated in YT. In addition, the identified flavonoid metabolites were annotated using the Kyoto Encyclopedia of Genes and Genomes (KEGG) database, and key pathways that might regulate the biosynthesis of these flavonoid compounds were analyzed. These findings provide key information for research on flavonoids and their biosynthesis in *C. tinctoria* and will provide a theoretical basis for studying the herbal quality and origin of *C. tinctoria*.

## Introduction

Flavonoids, the main secondary metabolites in plants, are the most important quality-related compounds and are also considered one of the sources of the medicinal properties in many herbal medicinal plants (Falcone Ferreyra, Rius & Casati, 2012; Zhang, Liu & Ruan, 2017; Li et al., 2021). According to the oxidation degree of the central three carbons, such as whether it forms a ring or serves as the connection site of the B ring, these compounds can be divided into the following substructure types: flavonoids, flavonols, dihydroflavonoids, dihydroflavonoid alcohols, isoflavones, dihydroisoflavones, chalcones, orange ketones, flavans, anthocyanidins and diflavonoids (Badshah et al., 2021; Zaragozá et al., 2021). Interest in the beneficial health and pharmacological effects of flavonoids has greatly increased due to their potent antioxidant and free radical scavenging activities (Zhang et al., 2020; Li et al., 2020b). Studies have shown that flavonoids can inhibit the reproduction of a variety of pathogenic microorganisms, and many mechanisms for this effect have been proposed, such as inhibiting nucleic acid synthesis and biofilm formation and changing the membrane permeability (Xie et al., 2015). Flavonoids can prevent a variety of cancers by regulating cell apoptosis, inhibiting tumor growth, and protecting organs from oxidative damage and have significant effects on reducing the blood glucose levels and antioxidant activity; for example, flavonoids in artichoke extract can reduce blood glucose in normal and obese rats (Fantini et al., 2011; Sun et al., 2021; Mondal et al., 2021). Moreover, some flavonoids can inhibit bacterial growth by inactivating adhesion proteins or membrane transporters and changing the membrane permeability (Cardona et al., 2013; Kumar & Pandey, 2013).

*C. tinctoria*, produced in the Kunlun Mountains and called ‘Kunlun Xueju’ or ‘Kunlun snow chrysanthemum’, is a naturalized annual herbaceous plant belonging to the genus *Coreopsis* of the *Compositae* family (Wang et al., 2015). Snow chrysanthemum is distributed in the northern foothills of Mt. Kunlun in China, usually grows in some areas with an altitude of 3,000 m above the snow line and is widely planted in western China, particularly in Mt. Kunlun in Xinjiang (Shao et al., 2010). *C. tinctoria* is currently the only alpine plant comparable to *Echeveria laui Moran & Meyrán*. Pharmacological studies have shown that *C. tinctoria* possesses multiple activities, including antioxidant, antidiabetic, antihypertensive, and cytoprotective effects (Dias et al., 2012; Lan, Lin & Zheng, 2014; Jiang et al., 2016; Jiang et al., 2018). Studies have confirmed that the various effects of *C. tinctoria* on the human body are mainly attributed to its flavonoid-like polyphenols because this plant contains many types of flavonoids (Zhao et al., 2013; Shen et al., 2020; Zhang et al., 2022). Nevertheless, due to differences in altitude, humidity, the soil environment, and the planting techniques in different regions, the metabolic mechanism of flavonoids in *C. tinctoria* may be different, and thus the types and contents of flavonoids are different depending on the geographical origin conditions, which will affect the medicinal value of *C. tinctoria*. Research has confirmed that the types and content of flavonoids vary among different species and different varieties of the same species (Yonekura-sakakibara, Higashi & Nakabayashi, 2019; Mou et al., 2021).

To reveal the differences in the flavonoid metabolites of *C. tinctoria* from different regions, a targeted analysis of flavonoids was performed based on a UPLC-QTOF-MS technique with three typical *C. tinctoria* types. An experiment comparing samples of *C. tinctoria* collected from Yutian County (growing in highland areas at a high altitude of approximately 3,200 m), Tashkurgan Tajik (growing at a medium altitude of approximately 2,000 m) and Shaya County (growing in the plains at an altitude of approximately 1,000 m) through a metabolomics analysis was therefore designed. This study will provide useful data for evaluating the nutritional and medicinal value of *C. tinctoria* of different origins and will lay a theoretical foundation for researching the quality and origin of *C. tinctoria*.

## Materials and Methods

### Plant materials

The samples of *C. tinctoria* species were collected from three different locations; specifically, samples were collected from Yutian County, Hotan Region, Xinjiang (growing in highland areas at a high altitude of approximately 3,200 m), Tashkurgan Tajik Autonomous County, Kashgar Region, Xinjiang (growing at a medium altitude of approximately 2,000 m), and Shaya County, Aksu Region, Xinjiang (growing in the plains at an altitude of approximately 1,000 m). At the flowering stage, the flowers of three *C. tinctoria* varieties were cut and packed into 2 mL tubes named YT, TX and SY. Three replicates of each sample were set up, rapidly frozen in liquid nitrogen and stored at −80 °C until the samples were extracted.

### Sample preparation and extraction

The sample was freeze-dried, ground into powder (30 Hz, 1.5 min), and stored at −80 °C until needed. Twenty milligrams of powder was weighed and extracted with 0.5 mL of 70% methanol. Ten microliters of internal standard (4,000 nmol/L) was added to the extract as an internal standard (IS) for quantification. The extract was sonicated for 30 min and centrifuged at 12,000*g* and 4 °C for 5 min. The supernatant was filtered through a 0.22 μm membrane filter for further LC‒MS/MS analysis. The flavonoid contents were detected by MetWare (http://www.metware.cn/) based on the AB Sciex QTRAP 6500 LC‒MS/MS platform.

### UPLC conditions

The sample extracts were analyzed using a UPLC‒ESI‒MS/MS system (UPLC, ExionLC™ AD, https://sciex.com.cn/; MS, Applied Biosystems 6500 Triple Quadrupole, https://sciex.com.cn/) according to Huang et al. (2021). The analytical conditions were set according to those reported by Zhang et al. (2021): UPLC; column, Waters ACQUITY UPLC HSS T3 C18 (100 mm × 2.1 mm i.d., 1.8 µm); solvent system, water with 0.05% formic acid (A), acetonitrile with 0.05% formic acid (B). The gradient elution program was set as follows: 0–1 min, 10–20% B; 1–9 min, 20–70% B; 9–2.5 min, 70–95% B; 12.5–13.5 min, 95% B; 13.5–13.6 min, 95–10% B; and 13.6–15 min, 10% B. The flow rate was set to 0.35 mL/min, and the temperature was set to 40 °C. The injection volume was 2 μL.

### ESI-MS/MS conditions

Linear ion trap (LIT) and triple quadrupole (QQQ) scans were obtained using a triple quadrupole-linear ion trap mass spectrometer (QTRAP; API 6500 Q TRAP LC/MS/MS System) equipped with an ESI Turbo Ion-Spray interface, operated in the positive and negative ion modes and controlled using Analyst 1.6.3 software (Sciex, Toronto, Canada). The ESI source operation parameters were set according to those reported by Wang et al. (2022). Flavonoid data were collected and identified by scheduled multiple reaction monitoring (MRM, New York, NW, USA). Data acquisition was performed using Analyst 1.6.3 software (Sciex, Toronto, Canada) according to Wang et al. (2022). MultiQuant 3.0.3 software (Sciex, Toronto, Canada) was used to quantify all metabolites. The mass spectrometer parameters, including depolymerization potential (DP) and collision energy (CE), for individual MRM transitions were performed with further DP and CE optimization (Liang et al., 2022; Wang et al., 2022). A specific set of MRM transitions was monitored during each period based on the metabolites eluted during each period (Liang et al., 2022). Flavonoid contents were detected using MetWare (https://www.metware.cn/) based on the AB Sciex QTRAP 6500 LC‒MS/MS platform.

### Multivariate statistical analysis of flavonoid metabolites

#### Principal component analysis

Unsupervised PCA was performed across all samples using the log2-normalized metabolite expression levels. Unit variance scaling was performed with the data before unsupervised PCA. Partial least squares discriminant analysis (PLS-DA) and orthogonal partial least squares discriminant analysis (OPLS–DA) were used to predict the stability and reliability of the models.

#### Hierarchical cluster analysis and Venn diagram comparison

Heatmaps with dendrograms were used to present the HCA results from the samples and flavonoid metabolites, and the Pearson correlation coefficients (PCCs) between samples were analyzed and calculated using the cor function in R and presented as heatmaps. The R package pheatmap was used for both the HCA and PCC in this study. For HCA, the normalized signal intensities of flavonoid metabolites (scaled per unit variance) were visualized as a color spectrum.

#### Selection of differential flavonoid metabolites

Significantly regulated metabolites between groups were determined based on VIP ≥ 1 and absolute Log_2_FC (fold change) ≥ 1.0. VIP values were extracted from the OPLS–DA results, which were also determined through score plots and permutation plots, and were generated using the R package MetaboAnalystR. The data were log-transformed (log2) and mean centered before OPLS–DA. To avoid overfitting, a permutation test (200 permutations) was performed.

## Results

### Flavonoid metabolic profiling

**Figure 1:**
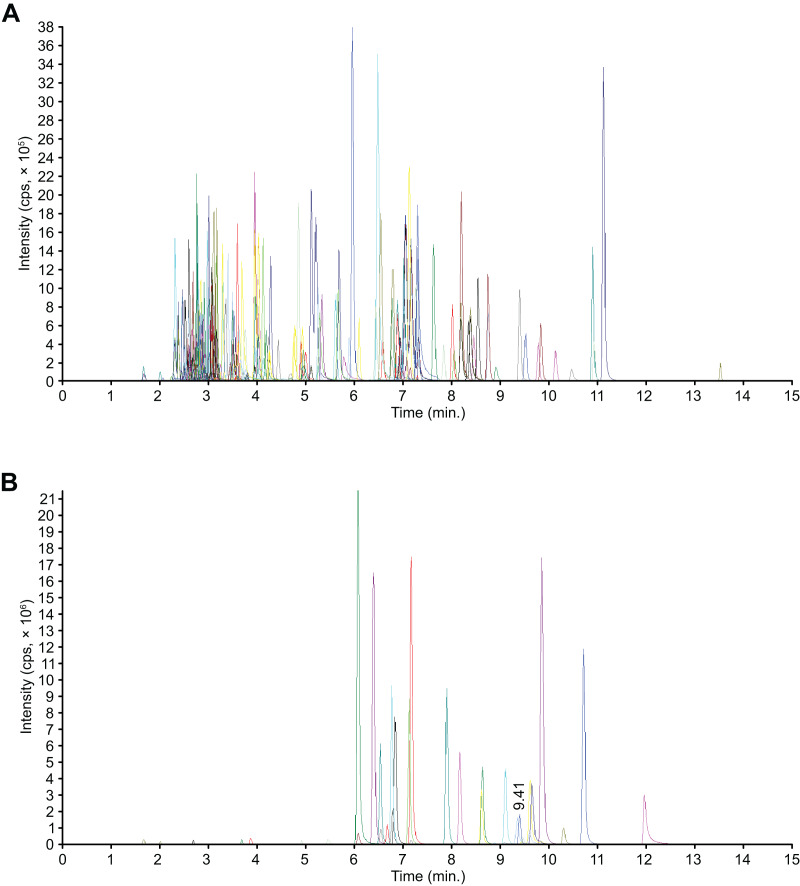
Mass spectrometry quality control (QC) total ion current (TIC) superposition diagram. (A) TIC in negative-ion MRM diagram. (B) TIC of the positive-ion MRM diagram.

Flavonoids are phenolic compounds and the major active ingredients found in the flowers of *C. tinctoria*. We performed a widely targeted metabolite analysis to comprehensively profile the flavonoids in the flowers of *C. tinctoria* based on UPLC–MS. Figure 1 show multipeak chromatograms of metabolites using multiple reaction monitoring (MRM). Based on the MetWare database, the metabolites of samples were determined qualitatively and quantitatively by UPLC-MS/MS. Under MRM mode, multipeak chromatograms of metabolites show detectable components. Every color indicated one metablite. Data files were opened by MultiaQuant software to integrate peaks and make calibrations. A total of 204 flavonoids, including 19 chalcones, seven flavanols, 19 flavanones, nine flavanonols, six flavone glycosides, 53 flavones, 37 flavonols, 24 isoflavanones, eight phenolic acids, three xanthones, three biflavonoids, one anthocyanin, and 15 unclassified flavonoids, were detected in flowers of *C. tinctoria* collected from three locations in the Xinjiang region at different altitudes (Table S1). The contents of flavonoid compounds in *C. tinctoria* varied significantly among regions.

### Flavonoid metabolite expression pattern analysis

**Figure 2:**
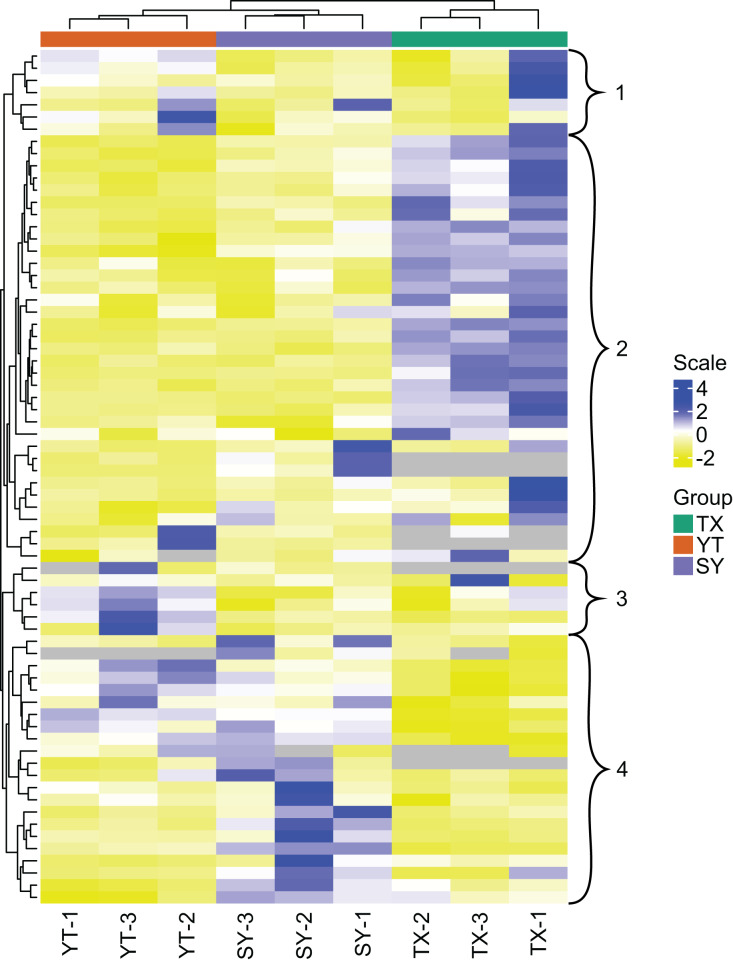
Clustering heatmap display of all of the detected flavonoid metabolites. Each sample has its own column, and each metabolite has its own row. The abundance of each metabolite is represented as a bar with a unique color.

All 204 detected flavonoid metabolites are illustrated by a heatmap (Fig. 2), and the different expression levels among the three varieties are displayed. A clustering of all the flavonoid metabolite expression contents revealed that the three *C. tinctoria* varieties were clearly different, particularly TX compared with SY and YT. The smallest differences in the flavonoid metabolite contents were found between SY and TX. The 204 flavonoid metabolites were divided into 13 groups: 19 chalcones, seven flavanols, 19 flavanones, nine flavanonols, six flavone glycosides, 53 flavones, 37 flavonols, 24 isoflavanones, eight phenolic acids, three xanthones, three biflavonoids, one anthocyanin, and 15 unclassified flavonoids.

The flavonoids in clusters 1 and 3 showed the highest levels of accumulation in YT collected from a high altitude of approximately 3,200 m and the lowest levels in SY found at an altitude of approximately 1,000 m (Fig. 2). The flavonoids in Cluster 2 accumulated to the highest levels in TX, which were collected from Tashkurgan Tajik Autonomous County, Kashgar Region, Xinjiang (growing at an altitude of approximately 2,000 m). Interestingly, the flavonoids in Cluster 2 were present at low levels in both YT and SY. The flavonoids in Cluster 4 were discovered in the highest levels in SY and at the lowest levels in TX.

### PCA of flavonoid metabolites identified from three *C. tinctoria* varieties

**Figure 3:**
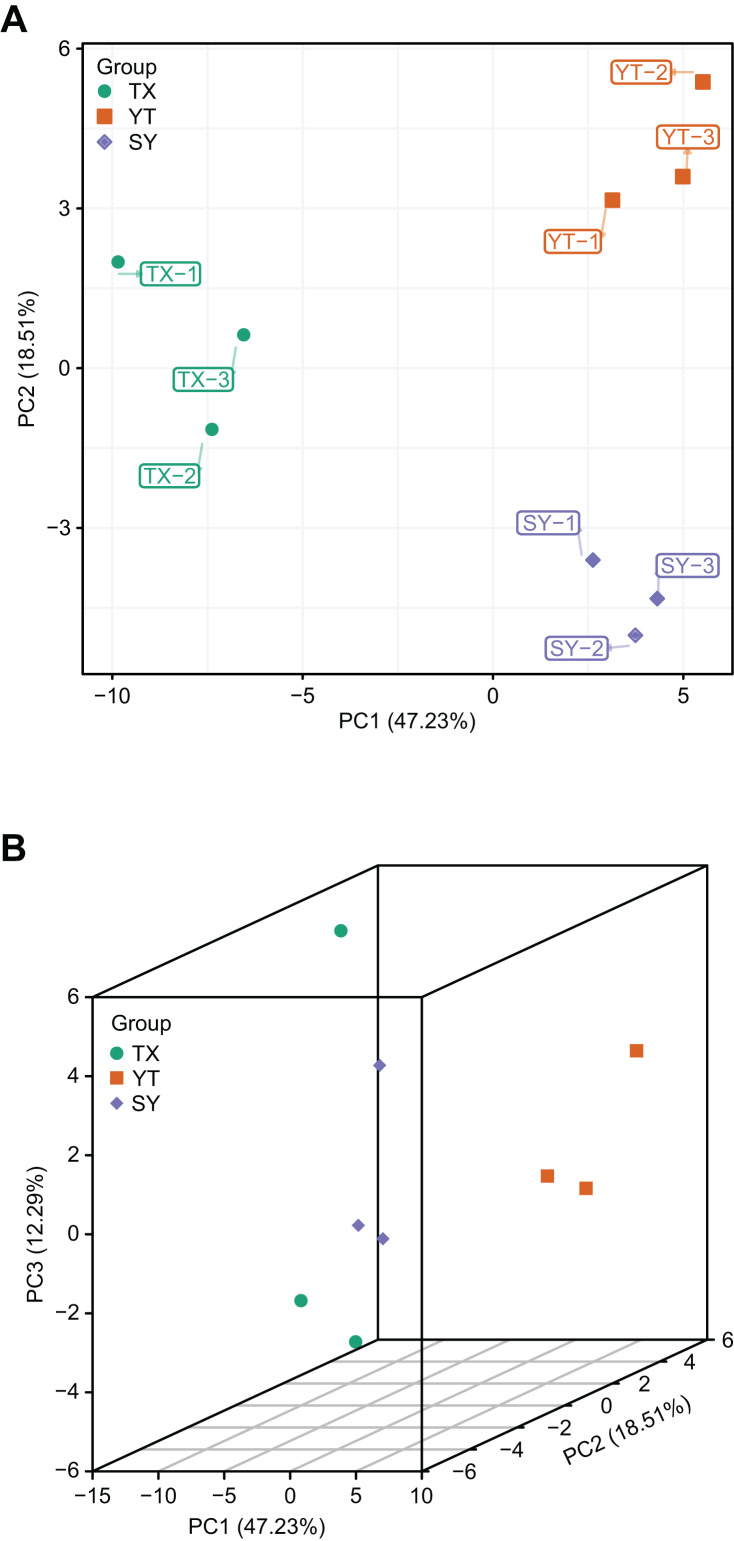
Differential metabolite analysis based on PCA. (A) 2D image and (B) 3D image.

A PCA of the samples (including QC samples) was performed to gain a preliminary understanding of the overall metabolic differences between groups and the magnitude of the variability between samples within groups. PCA is an innovative multivariate statistical analysis method that uses only a few principal components to illustrate the internal structure among multiple variables (Li et al., 2020b). The PCA results showed a trend of metabolome separation between groups, suggesting differences in the metabolome between sample groups. In the present study, three *C. tinctoria* varieties were used to analyze the repeatability of the samples subjected to the same treatments. The principal component scores showed that PC1 and PC2 explained 47.23% and 18.51% of the variability among the samples, respectively, and the total contribution rate reached 62.66% (Fig. 3). The three *C. tinctoria* variety samples were clearly separated and reproducible, and the replicates were compactly gathered, indicating the high reproducibility and scientific reliability of the data. Similarly, TX was significantly different from the other two *C. tinctoria* varieties, indicating that the metabolite profile of TX was notably distinguishable from those of the other two varieties and that the flavonoid composition of the three *C. tinctoria* varieties was highly divergent.

### Analysis of differential flavonoid metabolites by OPLS–DA

**Figure 4:**
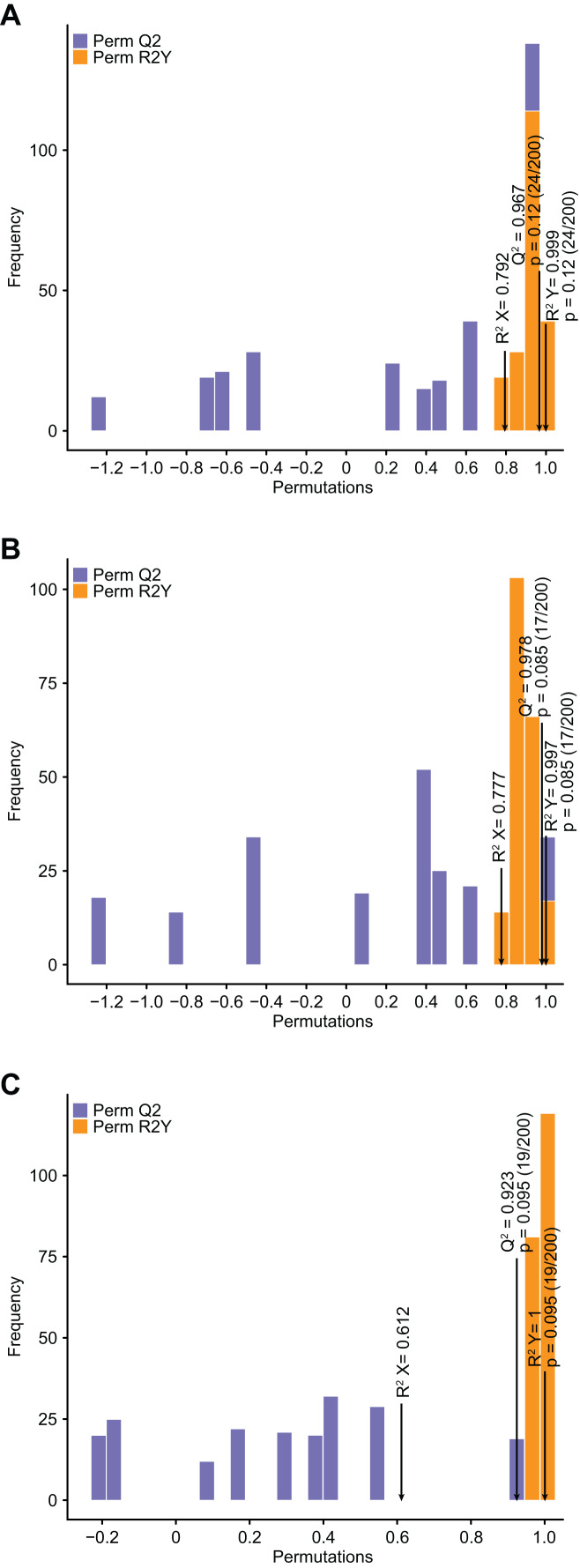
Differential flavonoid metabolite pairwise comparison OPLS–DA model plots. (A) OPLS–DA model plots for TX *vs*. YT. (B) OPLS–DA model plots for TX *vs*. SY. (C) OPLS–DA model plots for YT *vs*. SY.

Compared with PCA, OPLS–DA has the advantage of maximizing the distinction between groups and thus facilitates the search for differential metabolites (Li et al., 2020b). Q^2^ represents the predictive power of the model, and Q^2^ >0.9 is usually considered to indicate an excellent model (Li et al., 2020b). In the current study, pairwise comparison of *C. tinctoria* samples was performed using the OPLS–DA model to assess further differences between TX and YT (R^2^ X = 0.792, R^2^Y = 0.999, Q^2^ = 0.967; Fig. 4A), TX and SY (R^2^ X = 0.777, R^2^Y = 0.997, Q^2^ = 0.978; Fig. 4B), and YT and SY (R^2^ X = 0.612, R^2^Y = 1, Q^2^ = 0.923; Fig. 4C). In our study, the Q^2^ values of all the comparisons were close to 1, indicating that the models had high stability and reliability. In the OPLS–DA model score plot, these samples could be evidently separated, highlighting the large differences among the flavonoid metabolic profiles of *C. tinctoria* samples.

### Differential flavonoid metabolite identification and classification

**Figure 5:**
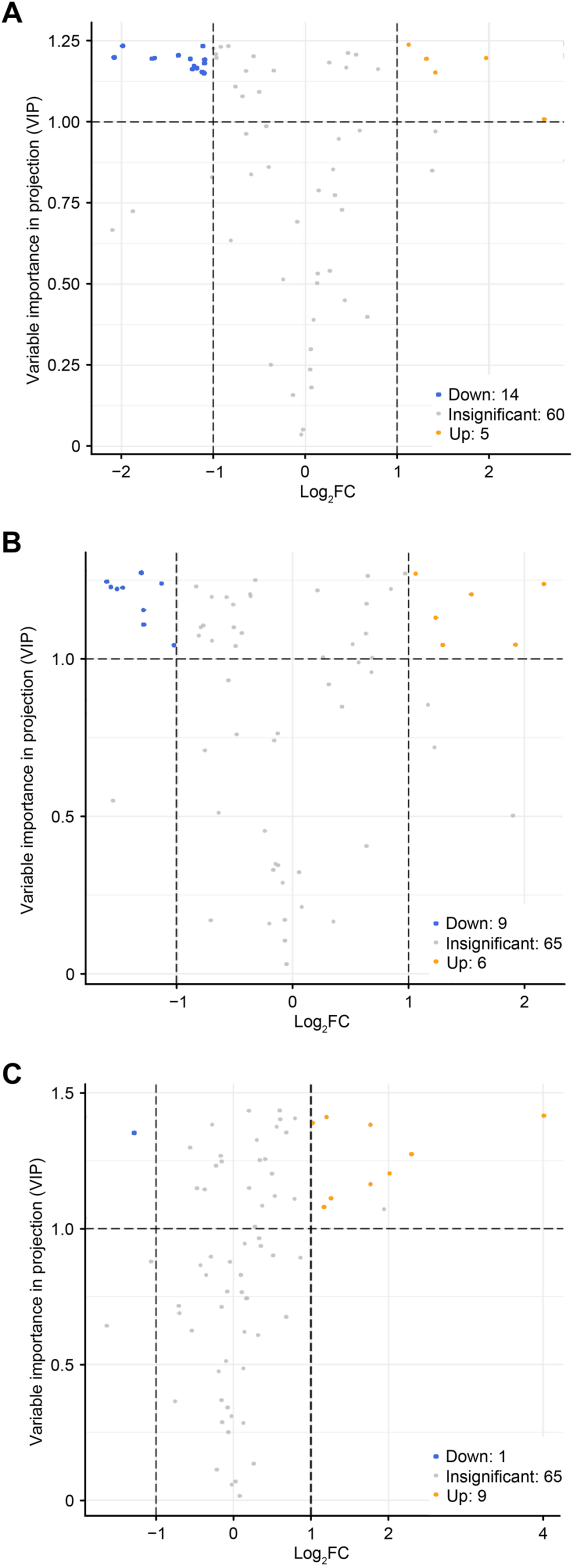
Volcano maps of differential metabolites. (A) TX *vs*. YT, (B) TX *vs*. SY and (C) YT *vs*. SY. Each dot represents a metabolite. The horizontal axis represents the log of the difference of a metabolite in the two samples, log2 (fold change).

**Figure 6:**
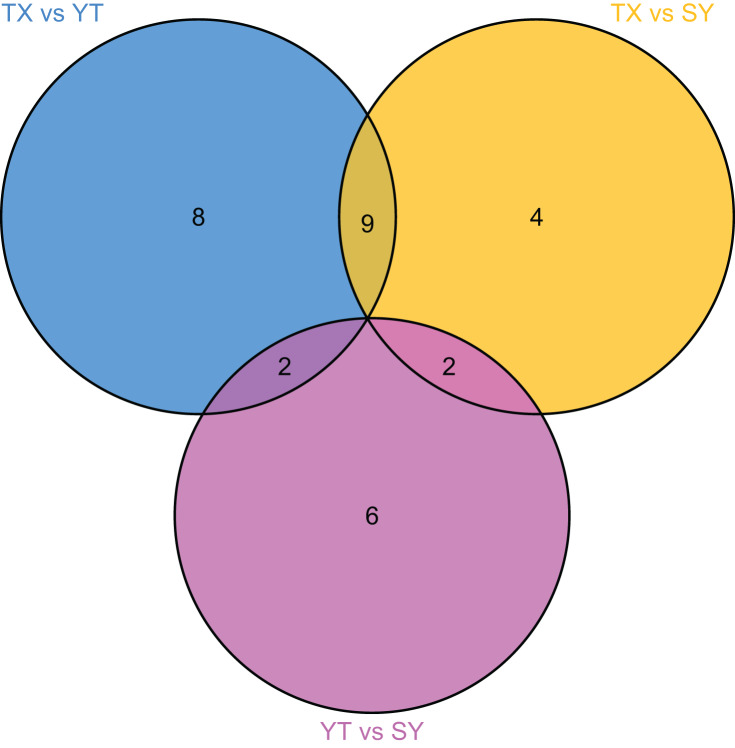
Venn diagram of differential flavonoid metabolites in the three *C. tinctoria* samples.

To accurately screen out the differential flavonoid metabolites among the three *C. tinctoria* varieties, we used a combination of the fold change in expression and variable importance in projection (VIP) values from the OPLS–DA model (Li et al., 2020b). Based on the OPLS–DA results, the VIP of the OPLS–DA model was calculated from the obtained multivariate data. The VIP value represents the intensity of the effect corresponding to the flavonoid metabolite differences between groups analyzed from the obtained multivariate data. Significant differential flavonoid metabolites were selected according to the following criteria: fold change ≥2 or ≤ 0.5 and VIP ≥ 1. Figure 5 displays the screening process for the differentials. The screening results of differential flavonoid metabolites from the three *C. tinctoria* varieties are illustrated using a Venn diagram (Fig. 6) and are displayed as a list of differential metabolites in Table 1. The comparisons identified 19 significantly different flavonoid metabolites between YT and TX (14 downregulated, five upregulated), 15 significantly different flavonoid metabolites between SY and TX (nine downregulated, six upregulated), and 10 significantly different flavonoid metabolites between SY and YT (one downregulated, nine upregulated) (Table S2).

**Table 1:** List of differential flavonoid metabolites in the three *C. tinctoria* samples.

Compound class	Index	Compound	Fold change		
			SY/TX	YT/TX	SY/YT
Flavones	Flavonoid_06	Diosmetin	-	0.46	-
	Flavonoid_59	Hispidulin	-	0.38	2.02
	Flavonoid_143	Jaceosidin	2.35	-	-
	Flavonoid_165	Homoplantaginin	0.41	0.43	-
	Flavonoid_167	Oroxin B	-	2.18	-
	Flavonoid_190	7,4′-Dihydroxyflavone	3.76	-	4.91
	Flavonoid_76	Sakuranetin	-	-	4.01
Flavonols	Flavonoid_43	Kaempferol	0.36	0.32	-
	Flavonoid_23	Quercetin	0.41	0.25	-
	Flavonoid_57	Rutin	2.45	-	-
	Flavonoid_58	Isorhamnetin	0.34	0.24	-
	Flavonoid_86	Avicularin	0.33	0.42	-
	Flavonoid_169	Quercetin 3-O-(6″-galloyl)-*β*-*D*-galactopyranoside Afzelin	0.49	0.47	-
	Flavonoid_175	Laricitrin	-	3.92	0.41
	Flavonoid_188	Quercimeritrin	-	0.44	-
	Flavonoid_194	Isorhamnetin 3-O-glucoside	0.41	0.47	-
	Flavonoid_197	Isorhamnetin-3-O	0.35	0.32	-
	Flavonoid_54	neohespeidoside Tiliroside	-	-	2.40
	Flavonoid_137	4,4′-Dimethoxychalcone	-	-	2.03
Chalcones	Flavonoid_03	Benzylideneacetophenone	4.49	-	3.41
	Flavonoid_200	Isosilybin	2.91	2.67	-
Flavanonols	Flavonoid_04	Taxifolin	-	2.01	-
	Flavonoid_56	Liquiritigenin	-	0.46	-
Flavanones	Flavonoid_12	Pinocembrin	2.09	-	-
	Flavonoid_108	Hesperetin	-	0.47	-
	Flavonoid_41	Poncirin	-	-	2.25
	Flavonoid_179	Daidzin	-	-	2.30
Isoflavanones	Flavonoid_32	Tectorigenin	-	2.50	-
	Flavonoid_63	Isomangiferin	-	0.43	-
Xanthone	Flavonoid_157	Schaftoside	0.46	-	-
Flavone	Flavonoid_74		-	-	3.41
glycosides					

**Note:**

Fold change values of ≥ 2 or ≤ 0.5 and VIP of ≥ 1 were considered to denote significant differences and were used as standards for screening metabolites. “-” Represents no significant difference.

The screening results of the differential flavonoid metabolites indicated that the greatest differences were found between TX and the other two *C. tinctoria* varieties, followed by SY and YT. No common differential flavonoid metabolites were found among the TX vs. YT, TX *vs*. SY and YT *vs*. SY comparison, which suggests that the flavonoid metabolite species causing the differences among TX, YT, and SY had a low degree of similarity.

### Differential flavonoid metabolite enrichment analysis

**Figure 7:**
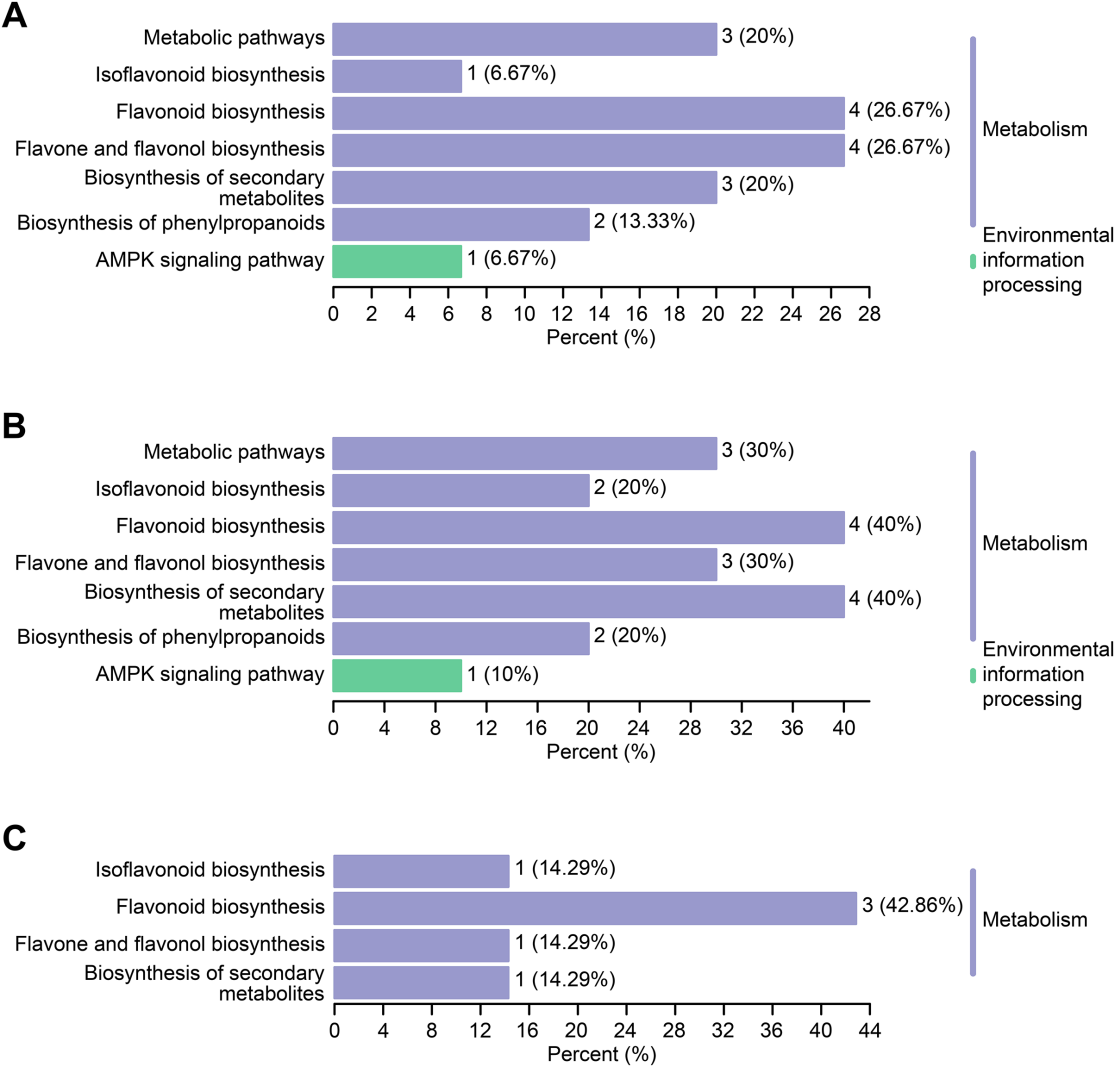
KEGG enrichment maps of the differential metabolites. (A–C) The KEGG enrichment maps among the comparison groups, including TX *vs*. YT, TX *vs*. SY and YT *vs*. SY, respectively.

Differential flavonoid metabolites can interact with each other in organisms to form distinct pathways. The differential flavonoid metabolites of *C. tinctoria* varieties were annotated using the Kyoto Encyclopedia of Genes and Genomes (KEGG) database. The abovementioned annotated metabolites of each comparison group are shown in Figs. 7A–7C . The KEGG enrichment analysis revealed that the significantly different flavonoid metabolites were distributed in pathways that included the biosynthesis of isoflavonoids (ko00943), flavonoids (ko00941), flavones and flavonols (ko00944), secondary metabolites (ko01110), phenylpropanoids (ko01061), and metabolic pathways (ko01100). Moreover, we noticed that one differential metabolite identified from the comparisons between TX and YT and between TX and SY was involved in the AMPK signaling pathway (ko04152).

## Discussion

*C. tinctoria* is a typical annual herb of the genus Coreopsis that is rich in medicinal components and possesses important medicinal and economic value (Shen et al., 2020). Medicinal phytochemical studies have confirmed that *C. tinctoria* contains active components such as flavonoids, organic acids, polysaccharides, alkaloids, terpenoids, phenylpropanoids, and volatile oil (Kumar & Pandey, 2013; Shen et al., 2020; Kim et al., 2021). Flavonoid biosynthesis is one of the most important secondary metabolism pathways in *C. tinctoria* (Kim et al., 2021). The flowers of *C. tinctoria* are rich in flavonoids, which play physiological roles that range from attracting pollinators to protection against biotic or abiotic stresses (Kim et al., 2021). Flavonoids acquired from medicinal plants are effective in scavenging free radicals, preventing cancer and diabetes, inhibiting inflammation, and preventing neurological and cardiovascular diseases (Jiang et al., 2015; Shen et al., 2020). Moreover, due to their different origins or genotypes, the medicinal ingredients in medicinal plants are bound to have different properties (Zhou et al., 2019; Wang et al., 2018a; Tan et al., 2021). In our study, the flavonoid contents in flowers of *C. tinctoria* collected from three locations in the Xinjiang region at different altitudes were analyzed. A total of 204 flavonoids, including 19 chalcones, seven flavanols, 19 flavanones, nine flavanonols, six flavone glycosides, 53 flavones, 37 flavonols, 24 isoflavanones, eight phenolic acids, three xanthones, three biflavonoids, one anthocyanin, and 15 unclassified flavonoids, were detected in the flowers of *C. tinctoria*. Compared with other medicinal plants, *C. tinctoria*, as a medicinal and food plant, has a higher abundance of flavonoid compounds than *Dendrobium officinale* (Zuo et al., 2020), *Morus alba* L. (Li et al., 2020a) and *Scutellaria baicalensis* (Wang et al., 2018b). For example, 135 flavonoid compounds and the maximum number of the metabolites in *D. officinale* stems were identified by UPLC–MS/MS metabolomics (Zuo et al., 2020), and Li et al. (2020a) reported 44 flavonoid compounds in mulberry leaves. A total of 56 flavonoids, including 42 flavones, two flavonols, nine flavanones, one flavonol, one chalcone, and one biflavonoid, have been isolated from *Scutellaria baicalensis* (Wang et al., 2018b). The abundance of flavonoid compounds may be responsible for the significant medicinal value of *C. tinctoria* from Mt. Kunlun. Therefore, *C. tinctoria* could be a more promising medicinal raw material and could be used as a new chrysanthemum functional tea (Wang et al., 2015; Jiang et al., 2018). Furthermore, further cultivation of *C. tinctoria* is of great significance to meet the different nutritional and healthcare needs of the population.

*C. tinctoria*, produced in Mt. Kunlun, called ‘Kunlun Xueju’, is grown in harsh plateau environments with limited production (Shao et al., 2010; Wang et al., 2015). Different geographical conditions, due to differences in altitude, humidity, soil environment, and climatic characteristics, may lead to the different metabolic mechanisms of the flavonoid compounds in plants (Yonekura-sakakibara, Higashi & Nakabayashi, 2019; Yu et al., 2020). Therefore, three samples of *C. tinctoria* were collected from three locations in the Xinjiang region at different altitudes, and the samples were collected from Yutian County, Hotan Region, Xinjiang (growing at an altitude of approximately 3,200 m), Tashkurgan Tajik Autonomous County, Kashgar Region, Xinjiang (growing at an altitude of approximately 2,000 m), and Shaya County, Aksu Region, Xinjiang (growing at an altitude of approximately 1,000 m). Fifteen significantly different metabolites were identified from the comparison of SY with TX (nine downregulated and six upregulated), and 19 significantly different metabolites were obtained from the comparison of YT with TX (14 downregulated and five upregulated). The analysis of the differential flavonoid metabolites identified from the TX *vs*. YT, TX vs. SY and YT vs. SY comparisons suggested that the metabolite profile of TX was notably distinguishable from those of the other two varieties. It is strongly believed that flavonoids in plant tissues can improve the stress resistance of plants and protect them from abiotic stresses, such as UV-B radiation and drought (Harborne & Williams, 2000; Xia et al., 2022). A previous study reported that flavonoids may function as antioxidants in response to excessive light exposure (Tattini et al., 2004). During *C. tinctoria* growth, the differences in climate conditions and the time of exposure to external environmental stress, such as light, may be one of the reasons for the higher accumulation of more flavonoids in TX than in the other varieties.

Moreover, most *C. tinctoria* on the market are planted artificially, and their quality is thus uneven. Flavonoids were used to evaluate the medicinal and edible plant quality based on the medicinal effectiveness in a previous study (Cao et al., 2019; Yu et al., 2016) due to the physiological functions of the flavonoid compounds. As a medicinal and edible plant of great economic value, the flavonoid components of *C. tinctoria* of different origins show indispensable quality characteristics, but there is little information available. Studies have shown that the content of flavonoids varies among different populations and different varieties of the same species (Yu et al., 2020; Mou et al., 2021). In the current study, seven flavonoid metabolites, namely, homoplantaginin, kaempferol, quercetin, isorhamnetin, avicularin, quercetin 3-O-(6′-galloyl)-*β*-*D*-galactopyranoside and isorhamnetin 3-O-glucoside, were highly accumulated only in TX and were found at low levels in YT and SY, (Table 1). 7,4′-Dihydroxyflavone and 4,4′-dimethoxychalcone were highly accumulated only in SY, and both were expressed at low levels in YT and TX; additionally, afzelin was specifically expressed at high levels in YT (Table 1). No common differential flavonoid metabolites were found among the three comparisons (TX *vs*. YT, TX *vs*. SY and YT *vs*. SY), which indicates a low degree of similarity among the flavonoid metabolic pathways responsible for the differences among TX, YT, and SY. The flavonoid contents of TX were significantly different from those of YT and SY. The TX origin has unique qualities that might make it the best cultivation region for *C. tinctoria*. These results suggest that the specific highly accumulated metabolites in each sample can be used as candidate substances to identify the origin of *C. tinctoria*.

The types and contents of flavonoid metabolites varied greatly among *C. tinctoria* species of different origins and at different altitudes, and each variety of a different origin accumulated its own unique flavonoid metabolites, which might provide these plants with potential health functions and medical value. Further KEGG enrichment analysis revealed that the differential flavonoid metabolites were distributed in pathways that included the biosynthesis of isoflavonoids, flavonoids, flavones and flavonols, secondary metabolites, phenylpropanoids, and metabolic pathways. Eleven metabolites were enriched in the flavonoid synthesis pathway (Fig. 7), which was also the pathway with the most enriched metabolites identified from the three comparison groups. Combined with the entire regulation networks of flavonoid metabolism, these findings might provide a theoretical basis for researching the medicinal quality of *C. tinctoria* and selecting the best origin.

## Conclusion

In the current study, we innovatively identified 204 flavonoid compounds in the flowers of *C. tinctoria* and then identified 31 differential flavonoid metabolites among flowers of *C. tinctoria* collected from three locations at different altitudes. Further analysis of these 31 significantly different flavonoid compounds revealed that specific highly accumulated flavonoid compounds were found in *C. tinctoria* flowers from each region, such as afzelin in YT and 7,4′-dihydroxyflavone in SY. Seven flavonoid metabolites, namely, homoplantaginin, kaempferol, quercetin, isorhamnetin, avicularin, quercetin 3-O-(6′-galloyl)-*β*-*D*-galactopyranoside and isorhamnetin 3-O-glucoside, were highly accumulated only in TX and were found at low levels in YT and SY. Furthermore, the KEGG database was used for pathway annotation of the differential flavonoid compounds, and key pathways that might regulate the biosynthesis of these flavonoid compounds were identified. This project provides preliminary insights into the study of flavonoid compounds and their biosynthesis in *C. tinctoria*. The differential flavonoid metabolites found in this study can be used as biomarkers to identify the origin and quality of *snow chrysanthemum*. These results will also lay a theoretical foundation for researching the medicinal quality and origin of *C. tinctoria*.

## Supplemental Information

10.7717/peerj.14580/supp-1 Supplemental Information 1 204 flavonoid compounds of *Coreopsis tinctoria* flower.

10.7717/peerj.14580/supp-2 Supplemental Information 2 Differential flavonoid compounds of TX vs YT TX vs SY and YT vs SY.

10.7717/peerj.14580/supp-3 Supplemental Information 3 Raw data.

## Additional Information and Declarations

### Data Availability

The following information was supplied regarding data availability: The raw data is available in the Supplemental File.
